# Tolerance and adaptive evolution of triacylglycerol-producing *Rhodococcus opacus* to lignocellulose-derived inhibitors

**DOI:** 10.1186/s13068-015-0258-3

**Published:** 2015-05-13

**Authors:** Kazuhiko Kurosawa, Josephine Laser, Anthony J Sinskey

**Affiliations:** Department of Biology, Massachusetts Institute of Technology, 77 Massachusetts Avenue, Cambridge, MA 02139 USA; Engineering Systems Division, Massachusetts Institute of Technology, 77 Massachusetts Avenue, Cambridge, MA 02139 USA; Present address: Institute of Biotechnology, Department Applied and Molecular Microbiology Berlin University of Technology, Gustav-Meyer-Allee 25, Berlin, D-13355 Germany

**Keywords:** Triacylglycerol, *Rhodococcus opacus*, Lignocellulose-derived inhibitor, Tolerance, Adaptation, Lignocellulosic fuel, Lipid-based biofuel

## Abstract

**Background:**

Lignocellulosic biomass has been investigated as a renewable non-food source for production of biofuels. A significant technical challenge to using lignocellulose is the presence of microbial growth inhibitors generated during pretreatment processes. Triacylglycerols (TAGs) are potential precursors for lipid-based biofuel production. *Rhodococcus opacus* MITXM-61 is an oleaginous bacterium capable of producing large amounts of TAGs on high concentrations of glucose and xylose present in lignocellulosic hydrolysates. However, this strain is sensitive to ligonocellulose-derived inhibitors. To understand the toxic effects of the inhibitors in lignocellulosic hydrolysates, strain MITXM-61 was examined for tolerance toward the potential inhibitors and was subjected to adaptive evolution for the resistance to the inhibitors.

**Results:**

We investigated growth-inhibitory effects by potential lignocellulose-derived inhibitors of phenols (lignin, vanillin, 4-hydroxybenzaldehyde (4-HB), syringaldehyde), furans (furfural and 5-hydroxymethyl-2-furaldehyde), and organic acids (levulinic acid, formic acid, and acetic acid) on the growth and TAG production of strain MITXM-61. Phenols and furans exhibited potent inhibitory effects at a concentration of 1 g L^−1^, while organic acids had insignificant impacts at concentrations of up to 2 g L^−1^. In an attempt to improve the inhibitor tolerance of strain MITXM-61, we evaluated the adaptation of this strain to the potential inhibitors. Adapted mutants were generated on defined agar media containing lignin, 4-HB, and syringaldehyde. Strain MITXM-61^SHL33^ with improved multiple resistance of lignin, 4-HB, and syringaldehyde was constructed through adaptive evolution-based strategies. The evolved strain exhibited a two- to threefold increase in resistance to lignin, 4-HB, and syringaldehyde at 50% growth-inhibitory concentrations, compared to the parental strain. When grown in genuine lignocellulosic hydrolysates of corn stover, wheat straw, and hardwood containing growth inhibitors, strain MITXM-61^SHL33^ exhibited a markedly shortened lag phase in comparison with that of strain MITXM-61.

**Conclusion:**

This study provides important clues to overcome the negative effects of inhibitors in lignocellulosic hydrolysates on TAG production of *R. opacus* cells. The findings can contribute to significant progress in detoxified pretreatment of hydrolysates and development of more efficient strains for industrial TAG fermentations of *R. opacus* using lignocellulosic biomass.

**Electronic supplementary material:**

The online version of this article (doi:10.1186/s13068-015-0258-3) contains supplementary material, which is available to authorized users.

## Background

The development of technologies capable of producing substitutes for petroleum-based fuels from lignocellulosic biomass has been extensively investigated [1,2]. Triacylglycerols (TAGs) are potential precursors to produce lipid-based biofuels such as biodiesel and hydrocarbon fuels [3-5], and are widely distributed in plants, animals, algae, and microorganisms [6,7]. Oleaginous microorganisms that utilize a variety of carbon substrates provide advantages for TAG production from renewable non-food resources such as lignocellulosic biomass [8].

Lignocellulose is composed of lignin and carbohydrate polymers of cellulose and hemicellulose, whose relative proportion depends on their biomass sources [9,10]. The hydrolysis of lignocellulose into fermentable sugars is essential for effective assimilation of the carbon feedstock by microorganisms [11,12]. During the conventional pretreatment and hydrolysis of lignocellulose with dilute acid, the liberation of the monomeric sugars is accompanied by the generation of by-products [13,14]. These by-products, and some materials present in the lignocellulose, are inhibitory to microbial metabolism, causing low yields and productivities in the fermentation processes [15-17]. Toxicity of lignocellulose-derived materials can be a critical obstacle in the potentiality of biotechnological conversions of lignocellulosic biomass to biofuels [18,19]. The major inhibitory materials include furans (for example, furfural and HMF) derived from elimination reactions of pentose and hexose sugars, phenols (for example, syringaldehyde, vanillin, 4-HB, and soluble lignin (lignosulfonates)) originating from the degrading of the phenolic polymer lignin, and organic acids (for example, acetic, formic, and levulinic acids) deriving from the de-acetylation of hemicellulose or generated from the breakdown of furfural and 5-(hydroxymethyl)-2-furaldehyde (HMF) [20-22]. The composition and content of these inhibitors present in lignocellulosic hydrolysates depends on the type of raw materials used and the operational conditions during pretreatment and hydrolysis processes [23,24]. It is also known that inhibitory effects on microbial cell growth and metabolism vary with their concentration and the microorganisms [25,26]. Extensive studies have recently been performed on the effect of the lignocellulose-derived inhibitors in ethanol-fermenting microorganisms and oleaginous yeasts like *Corynebacterium glutamicum* [27], *Escherichia coli* [28], *Zymomonas mobilis* [29], *Cryptococcus curvatus* [30], *Pichia stipites* [31], *Rhodosporidium toruloides* [32], *Saccharomyces cerevisiae* [33], and *Trichosporon fermentas* [34]. To address the negative effects of inhibitors, several strategies have been investigated. One possibility is to exploit detoxification processes, including physical, chemical, or biological methods, prior to the fermentation [35-38]. However, inhibitor detoxification tends to be complicated and causes an increase of production cost [39,40]. Utilizing a combination of inhibitor-tolerant strains with desired properties for detoxification of lignocellulosic hydrolysates could be a more cost-effective approach for the industrial-scale fermentations [19,41]. Much effort has been devoted over the past decade to obtaining production strains with improved inhibitor tolerance. Microbial tolerance to these inhibitors has been further improved by genetic and evolutionary engineering strategies [19,42]. Substantial progress has been made in minimizing the effects of the inhibitors on the performance of yeast strains. *S. cerevisiae* has been engineered for increased tolerance to fermentation inhibitors by overexpressing genes encoding enzymes conferring improved tolerance to phenolics, furans, and organic acids, or by overexpressing a transcription factor and multidrug-resistance proteins [43-46]. Evolution-based strategies have also been attempted to improve inhibitor tolerance of *S. cerevisiae* [47-49]. Some studies suggest that the use of adaptive evolution to generate inhibitor-tolerant strains is a more effective method, as compared to the genetic engineering approach [50,51].

*Rhodococcus opacus* PD630 produces significant amounts of intracellular TAGs, composed primarily of C16 and C18 series of long chain fatty acids, which are similar to those of vegetable-derived TAGs [52,53]. In addition, this strain is able to accumulate these TAGs in batch-cultivations containing high concentrations of glucose [54]. Although it does not assimilate the xylose that tends to be abundantly present in lignocellulosic hydrolysates, we have recently enabled TAG production from xylose in *R. opacus* cells by heterologously expressing two genes, *xylA* and *xylB* [55]. More recently, through an adaptive evolution strategy, we have constructed a more high-potency xylose-fermenting strain (MITXM-61) that is capable of simultaneously and completely utilizing mixed sugars of xylose and glucose at high concentrations from corn stover hydrolysate and producing 15.9 g L^−1^ of TAGs with a productivity of 0.133 g L^−1^ h^−1^, corresponding to 54% of the cell dry weight [56]. The evolved strain possesses the potential to formulate a new manufacturing paradigm for developing hydrocarbon-based biofuels from lignocellulosic biomass. However, *R. opacus*, like many bacteria and yeast, is highly susceptible to lignocellulose-derived inhibitors, as our previous studies have shown that *R. opacus* had the long lag phase when lignocellulosic hydrolysates were used for the TAG fermentation [55-57].

Up to now, very little was known about the fermentation performance of the genus *Rhodococcus* in the presence of lignocellulose-derived inhibitors. In more recent results, only some physiological effects of several inhibitors on the growth and TAG production of *R. opacus* PD630 have been reported [58]. To date, no studies have been devoted to generate the inhibitor-tolerant *Rhodococcus* strains. The objective of this study was to provide the fundamental understanding necessary for TAG production on lignocellulosic hydrolysates with *R. opacus*. Accordingly, we examined the effects of nine common lignocellulose-derived inhibitors on the cell growth and TAG production by the xylose-utilizing *R. opacus* strain, MITXM-61 strain. Then, based on their inhibitory performance, we investigated to generate the strain with improved tolerance against the inhibitors using an evolutionary adaptation approach.

## Results

### Effects of individual lignocellulose-derived inhibitors on the growth and TAG production of *R. opacus* MITXM-61

**Figure 1:**
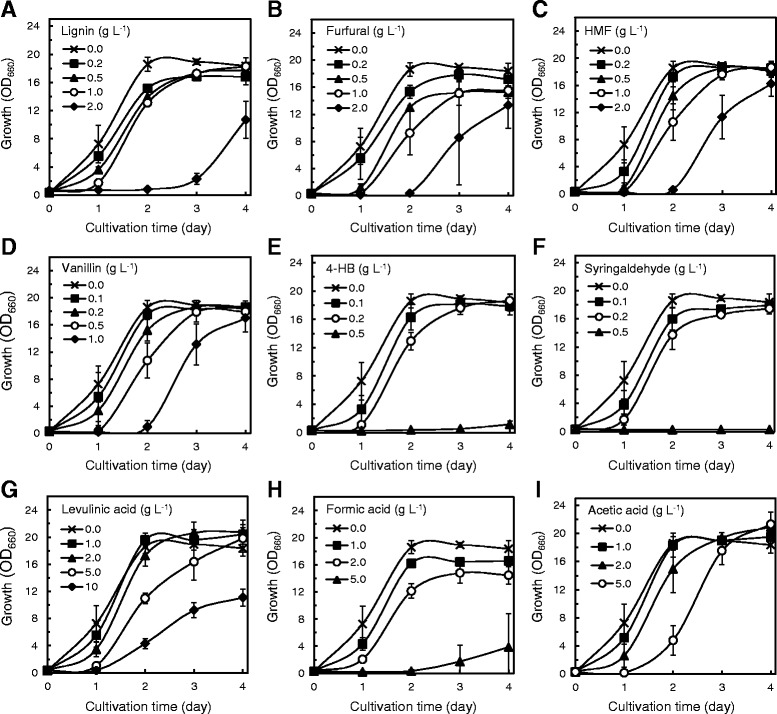
Growth profiles of strain MITXM-61 in the presence of common lignocellulose-derived inhibitors. The strain was grown in defined media containing 16 g L^−1^ glucose supplemented with different concentrations of lignin **(A)**, furfural **(B)**, 5-hydroxymethyl-2-furaldehyde (HMF) **(C)**, vanillin **(D)**, 4-hydroxybenzaldehyde (4-HB) **(E)**, syringaldehyde **(F)**, levulinic acid **(G)**, formic acid **(H),** and acetic acid **(I)**. Values and error bars represent the mean and the standard deviation of triplicate experiments.

In order to validate the elemental effects of lignocellulose-derived inhibitors on the cell growth and TAG production of *R. opacus*, nine representative inhibitors, commonly found in lignocellulosic hydrolysates, were included in a defined medium at various concentrations. Strain MITXM-61 was cultivated in the media containing 16 g L^−1^ glucose supplemented with lignin (0 to 2.0 g L^−1^), furfural (0 to 2.0 g L^−1^), HMF (0 to 2.0 g L^−1^), vanillin (0 to 1.0 g L^−1^), 4-hydroxybenzaldehyde (4-HB) (0 to 0.5 g L^−1^), syringaldehyde (0 to 0.5 g L^−1^), levulinic acid (0 to 10 g L^−1^), formic acid (0 to 5.0 g L^−1^), or acetic acid (0 to 5.0 g L^−1^) as individual inhibitors. The phenolic lignins, vanillin, 4-HB, and syringaldehyde exhibited drastic inhibitory effects on *R. opacus* strain MITXM-61 (Figure 1A,D,E,F). The presence of 0.5 g L^−1^ of 4-HB and syringaldehyde in the media resulted in nearly complete inhibition of cell growth. When vanillin and lignin were added at concentrations of 1.0 g L^−1^, the cell growth after 2 days of cultivation dropped by 95 (±5) % and 29 (±11) %, respectively, from that of the control. The furans of furfural and HMF had similar inhibition profiles and appeared to be slightly less toxic than 4-HB, syringaldehyde, and vanillin (Figure 1B,C). At the initial concentration of 1.0 g L^−1^, growth inhibition after 2 days of cultivation was observed to be 51 (±15) % by furfural and 43 (±12) % by HMF. The three organic acids demonstrated slight inhibitory effects on the growth up to 2.0 g L^−1^ (Figure 1G,H,I). However, the addition of 5.0 g L^−1^ of levulinic acid, formic acid, or acetic acid to the media caused 41 (±4) %, 98 (±1) %, and 74 (±11) % growth inhibition, respectively, after 2 days of cultivation as compared to the control. Among the organic acids tested, when levulinic acid or acetic acid at a concentration of 2.0 g L^−1^ were added to the medium, the maximum cell densities during 4 days of cultivation were increased by 10 (±3) % and 11 (±4) %, respectively, as compared with those of the control, suggesting that the strain may be capable of utilizing the organic acids as carbon sources. The half maximal inhibitory concentrations (IC_50_) following 2-day exposure in the growth of *R. opacus* MITXM-61 for the lignocellulose-derived inhibitors are summarized in Table 1.

**Table 1:** **Effects of common lignocellulose-derived inhibitors on the growth of** ***R*** **.** ***opacus*** **strains**

**Inhibitor**	**IC**_**50**_ **, g L** ^**−1**^	
	**MITXM-61**	**MITXM-61** ^**SHL33**^
Lignin	1.2 (±0.04)	3.5 (±0.26)
Furfural	1.0 (±0.29)	1.0 (±0.12)
HMF	1.1 (±0.23)	1.2 (±0.06)
Vanillin	0.55 (±0.09)	0.60 (±0.06)
4-HB	0.28 (±0.03)	0.73 (±0.03)
Syringaldehyde	0.30 (±0.03)	0.57 (±0.01)
Levulinic acid	5.8 (±0.72)	5.4 (±0.20)
Formic acid	2.7 (±0.20)	2.6 (±0.50)
Acetic acid	3.3 (±0.78)	4.2 (±0.36)

The strain was cultivated in defined media containing 16 g L^−1^ glucose supplemented with different concentrations of the inhibitors for 2 days. Inhibitory concentrations of inhibitors at 50% (IC_50_) were estimated from the growth profile. Data are results of triplicate experiments, ±s.d.

**Figure 2:**
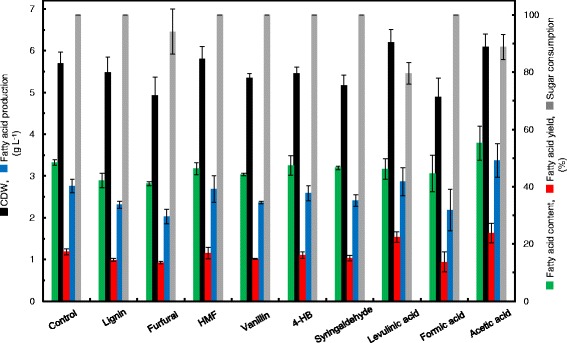
TAG production as fatty acids of strain MITXM-61 in the presence of common lignocellulose-derived inhibitors. The strain was grown in defined media containing 16 g L^−1^ glucose supplemented with 1 g L^−1^ lignin, 1 g L^−1^ furfural, 1 g L^−1^ HMF, 0.5 g L^−1^ vanillin, 0.2 g L^−1^ 4-HB, 0.2 g L^−1^ syringaldehyde, 5 g L^−1^ levulinic acid, 2 g L^−1^ formic acid, and 5 g L^−1^ acetic acid for 4 days in Figure 1. Values and error bars represent the mean and the standard deviation of triplicate experiments.

We also determined TAG production and the fatty acid composition profile of TAGs from MITXM-61 cells growing for 4 days in the presence of the nine individual inhibitors at their concentrations bringing about the growth inhibition of more than 90% after 1 day of cultivation relative to the control (Figure 2 and Table 2). When the strain was cultivated in the defined medium containing glucose in the absence of an inhibitor (the control), the fatty acid content (as percent of CDW), fatty acid production, and fatty acid yield (glucose to fatty acids) were 48 (±0.9) %, 2.7 (±0.2) g L^−1^, and 17 (±1.0) %, respectively. Although there were no significant differences in the TAG production and the fatty acid composition profile between the control and the seven inhibitors of lignin, furfural, HMF, vanillin, 4-HB, syringaldehyde, and formic acid, it was observed that levulinic acid and acetic acid affected the TAG production of the cells. Acetic acid improved the cell mass production and cell performance as lipid content. The fatty acid content, fatty acid production, and fatty acid yield of strain MITXM-61 were 55 (±11) % of cell dry weight (CDW), 3.4 (±0.5) g L^−1^, and 24 (±5.4) %, respectively, in the presence of 5.0 g L^−1^ acetic acid. Levulinic acid enhanced the cell mass production and altered the fatty acid composition in the TAG molecule. In the presence of 5.0 g L^−1^ levulinic acid, the fatty acid content, fatty acid production, and fatty acid yield were 46 (±3.6) % of CDW, 2.9 (±0.3) g L^−1^ and 22 (±1.8) %, respectively. The fatty acid composition of TAGs accumulated under the conditions with levulinic acid present in the culture media consisted mainly of C15 and C17 series fatty acids. In contrast, *Rhodococcus* grown on the media supplemented lignin, furfural, HMF, vanillin, 4-HB, syringaldehyde, formic acid, and acetic acid generated TAGs constituting primarily of C16 and C18 series fatty acids, and the fatty acid composition profiles were similar to those from the media containing glucose in the absence of the inhibitor.

**Table 2:** **Fatty acid composition as % of total fatty acids (g g** ^**−1**^ **) of TAGs from cells**

**Fatty acid species**				**Lignocellulose-derived inhibitor**						
	**Control**	**Lignin**	**Furfural**	**HMF**	**Vanillin**	**4-HB**	**Syringaldehyde**	**Levulinic acid**	**Formic acid**	**Acetic acid**
Myristic acid (C14:0)	2.0 (±0.1)	2.0 (±0.1)	1.9 (±0.1)	1.9 (±0.1)	1.5 (±0.4)	1.9 (±0.1)	2.0 (±0.1)	1.0 (±0.1)	2.0 (±0.2)	2.0 (±0.1)
Pentadecanoic acid (C15:0)	5.2 (±0.5)	4.4 (±0.5)	7.1 (±0.1)	5.5 (±0.4)	5.7 (±0.4)	5.6 (±0.5)	5.5 (±0.5)	13.9 (±1.0)	8.9 (±0.8)	5.4 (±0.6)
Palmitic acid (C16:0)	28.9 (±0.5)	28.4 (±0.6)	29.4 (±0.4)	27.3 (±0.9)	26.5 (±0.4)	26.1 (±0.6)	25.1 (±0.6)	15.2 (±0.6)	23.2 (±0.6)	27.2 (±1.5)
Palmitoleic acid (C16:1)	10.2 (±0.4)	8.0 (±1.6)	9.6 (±0.5)	8.3 (±0.6)	7.5 (±1.4)	7.4 (±0.9)	8.3 (±0.6)	3.9 (±0.1)	8.6 (±0.5)	8.9 (±0.2)
Heptadecanoic acid (C17:0)	9.9 (±0.8)	10.5 (±0.8)	10.9 (±0.1)	11.9 (±0.1)	12.2 (±0.5)	11.9 (±0.1)	11.6 (±0.5)	19.2 (±0.6)	12.9 (±0.1)	11.0 (±1.0)
*cis*-10-Heptadecenoic acid (C17:1)	15.4 (±0.6)	14.0 (±0.8)	15.3 (±0.4)	15.7 (±0.9)	17.5 (±0.4)	16.5 (±0.6)	17.8 (±0.9)	30.3 (±1.1)	20.3 (±0.6)	16.2 (±1.9)
Stearic acid (C18:0)	5.0 (±0.2)	6.4 (±0.8)	4.7 (±0.6)	5.6 (±0.6)	4.7 (±0.6)	5.7 (±0.4)	4.6 (±0.5)	2.9 (±0.1)	3.9 (±0.1)	5.4 (±0.6)
Oleic acid (C18:1)	22.5 (±1.3)	25.3 (±1.3)	20.0 (±1.2)	22.5 (±0.9)	23.7 (±1.8)	23.9 (±1.8)	24.1 (±1.3)	12.4 (±0.5)	19.3 (±1.5)	23.0 (±1.4)
Linoleic acid (C18:2)	1.0 (±0.1)	1.0 (±0.2)	1.0 (±0.2)	1.1 (±0.3)	0.9 (±0.1)	1.1 (±0.2)	1.0 (±0.2)	1.2 (±0.1)	0.9 (±0.1)	1.0 (±0.2)

Grown in the presence of lignocellulose-derived inhibitors.

Strain MITXM-61 was cultivated in defined media containing 16 g L^−1^ glucose supplemented with 1 g L^−1^ lignin, 1 g L^−1^ furfural, 1 g L^−1^ HMF, 0.5 g L^−1^ vanillin, 0.2 g L^−1^ 4-HB, 0.2 g L^−1^ syringaldehyde, 5 g L^−1^ levulinic acid, 2 g L^−1^ formic acid, and 5 g L^−1^ acetic acid for 4 days in Figure 1. Data are results of triplicate experiments, ±s.d.

### Effects of levulinic acid and acetic acid on TAG production of *R. opacus* MITXM-61

In order to provide insights into the substrate assimilation, strain MITXM-61 was cultivated in modified defined media containing 2.0 g L^−1^ of levulinic acid and acetic acid as the sole carbon source. As shown in Table 3A, the strain was able to utilize both organic acids and accumulate intracellular TAGs. The kinetics of TAG production of strain MITXM-61 grown on levulinic acid alone and acetic acid alone were lower than those attained with glucose alone. The fatty acid composition of TAGs from the cells grown on acetic acid and glucose was quite similar, and the accumulated TAGs consisted primarily of C16:0, C18:1, and C17:1 (Table 3B). In contrast, the cells grown on levulinic acid generated mostly odd-carbon fatty acids, composed of C17:1, C15:0, and C17:0, in relative abundance accumulating to as much as 80% in the cells of the total fatty acids detected. As shown in Figure 2 and Table 2, taking the impact of glucose upon the organic acids into consideration, these data implied that levulinic acid and acetic acid might stimulate the TAG production of *R. opacus* and result in an increase of the TAG yield when added at low concentrations into the sugar-containing medium. In addition, the presence of levulinic acid in the medium could affect the fatty acid biosynthetic pathway of *R. opacus* and change the distribution of major fatty acids in the TAG molecule, facilitating the strain to synthesize odd-carbon chain fatty acids in high abundance and store them as TAGs.

**Table 3:** **TAG production of strain MITXM-61 on levulinic acid and acetic acid**

**A. Fermentation kinetics**					
**Carbon source**	**Growth, CDW**	**TAG production as fatty acids**		**Residual substrate**	**Fatty acid yield**
	**g L** ^**−1**^ **of culture**	**% CDW**	**g L** ^**−1**^ **of culture**	**g L** ^**−1**^ **of culture**	**%**
Levulinic acid	0.68 (±0.04)	38.1 (±3.1)	0.26 (±0.03)	0.05 (±0.03)	13.3 (±1.9)
Acetic acid	0.63 (±0.05)	34.1 (±3.4)	0.21 (±0.03)	0.00 (±0.00)	10.7 (±1.2)
Glucose	0.73 (±0.02)	42.3 (±2.2)	0.31 (±0.01)	0.00 (±0.00)	15.4 (±0.6)
**B. Fatty acid composition profile as % of total acids (g g** ^**−1**^ **) of TAGs**					
**Fatty acid species**			**Carbon source**		
		**Levulinic acid**	**Acetic acid**		**Glucose**
Myristic acid (C14:0)		1.0 (±0.0)	2.0 (±0.0)		1.8 (±0.2)
Pentadecanoic acid (C15:0)		23.0 (±1.0)	7.3 (±0.6)		5.5 (±0.6)
Palmitic acid (C16:0)		6.7 (±0.6)	23.3 (±1.2)		25.9 (±1.9)
Palmitoleic acid (C16:1)		2.3 (±0.6)	7.3 (±0.6)		8.5 (±0.5)
Heptadecanoic acid (C17:0)		21.3 (±0.6)	13.3 (±0.6)		11.3 (±1.2)
*cis*-10-Heptadecenoic acid (C17:1)		37.7 (±0.6)	20.0 (±1.0)		16.8 (±1.0)
Stearic acid (C18:0)		1.0 (±0.0)	4.3 (±0.6)		4.6 (±0.4)
Oleic acid (C18:1)		6.0 (±0.0)	21.7 (±0.6)		24.8 (±0.9)
Linoleic acid (C18:2)		1.2 (±0.2)	0.7 (±0.3)		1.0 (±0.3)

The strain was cultivated in modified defined media containing 2.0 g L^−1^ of levulinic acid, acetic acid, or glucose supplemented with 0.125 g L^−1^ (NH_4_)_2_SO_4_ for 3 days. Data are results of triplicate experiments, ±s.d.

### Combined effects of lignocellulose-derived inhibitors on the growth of *R. opacus* MITXM-61

**Figure 3:**
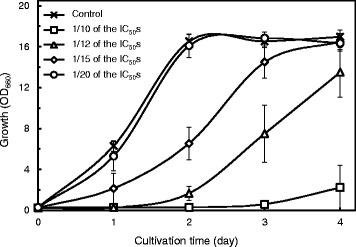
The combined effects of common lignocellulose-derived inhibitors on the growth of strain MITXM-61. The strain was grown in defined media in presence of the mixed nine inhibitors at 1/10, 1/12, 1/15, and 1/20 of the IC_50_ against strain MITXM-61 at the exposure duration of 2 days (1.2 g L^−1^ lignin, 1.0 g L^−1^ furfural, 1.1 g L^−1^ HMF, 0.55 g L^−1^ vanillin, 0.28 g L^−1^ 4-HB, 0.30 g L^−1^ syringaldehyde, 5.8 g L^−1^ levulinic acid, 2.7 g L^−1^ formic acid, and 3.3 g L^−1^ acetic acid) in each of inhibitors. Values and error bars represent the mean and the standard deviation of triplicate experiments.

Synergistic growth-inhibitory effects between various lignocellulose-derived materials have been reported [29,32]. We designed experiments to investigate the interactions among representative inhibitors on the cell growth of *R. opacus*. The combined effects of those inhibitors were tested in defined glucose-containing media mixed with nine lignocellulose-derived inhibitors at various concentrations, 1/10, 1/12, 1/15, and 1/20 of their respective IC_50_ against strain MITXM-61 (Table 1). When the nine inhibitors were added to the medium at a 1/10 concentration of their IC_50_ values, the relative cell density of strain MITXM-61 after 2 days of cultivation was predicted to be an approximate 50% growth inhibition as compared to the control, in view of the data obtained in experiments using individual inhibitors. If the actual experimental data exceeded the expected value, the inhibition by the nine inhibitors was defined as synergistic. As shown in Figure 3, after 2 days of cultivation, the cultural condition including 1/10 of the IC_50_ gave almost complete growth suppression, showing that the synergistic effect of combined inhibitors on repressing the growth of the cells was observed. The presence of the nine inhibitors at 1/12, 1/15, and 1/20 concentrations of their respective IC_50_ in the media led to 90 (±4.3) %, 60 (±9.6) %, and 3.9 (±3.5) % inhibitions, respectively, on the growth of strain MITXM-61.

### Generation of lignocellulosic inhibitor-tolerant *R. opacus* strains

**Figure 4:**
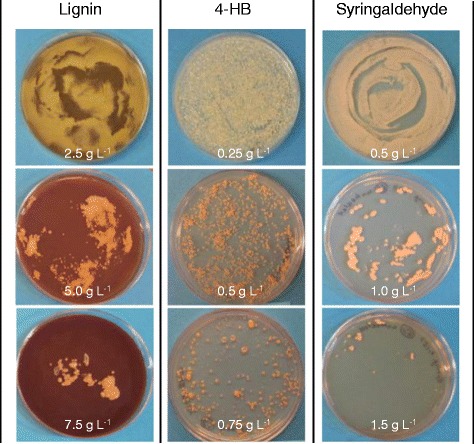
Colonies arising from plate cultures of strain MITXM-61 with lignocellulose-derived inhibitors. The bacterial cells were plated onto defined agar media containing 16 g L^−1^ glucose supplemented with 2.5, 5.0 and 7.5 g L^−1^ of lignin, 0.25, 0.5, and 0.75 g L^−1^ of 4-HB, or 0.5, 1.0 and 1.5 g L^−1^ of syringaldehyde, and incubated for 14 days.

**Figure 5:**
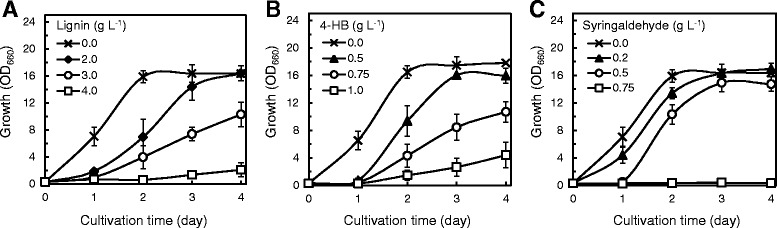
Growth profiles of *R. opacus* MITXM-61-derivatives in the presence of the lignocellulose-derived inhibitors. Strains MITXM-61^L33^ **(A)**, MITXM-61^H6^ **(B),** and MITXM-61^S1^ **(C)** were grown in defined media containing 16 g L^−1^ glucose supplemented with different concentrations of lignin, 4-HB, or syringaldehyde, respectively. Values and error bars represent the mean and the standard deviation of triplicate experiments.

We have successfully constructed *R. opacus* strains capable of effectively utilizing both xylose and glycerol to produce TAGs through adaptive evolution using electroporated cells [56,59]. In order to improve tolerance of *R. opacus* to the lignocellulose-derived inhibitors, the competent cells of strain MITXM-61 were treated by electroporation. The pulsed cells were spread on defined glucose-containing agar media supplemented with lignin (2.5 to 10 g L^−1^), furfural (2.5 to 10 g L^−1^), HMF (2.5 to 10 g L^−1^), vanillin (0.5 to 2.0 g L^−1^), 4-HB (0.25 to 2.0 g L^−1^), syringaldehyde (0.5 to 2.0 g L^−1^), levulinic acid (10 to 30 g L^−1^), formic acid (10 to 30 g L^−1^), and acetic acid (10 to 30 g L^−1^). After 14 days of cultivation, characteristic colonies were observed on the plates containing 5.0 to 7.5 g L^−1^ of lignin, 0.5 to 0.75 g L^−1^ of 4-HB, and 1.0 to 1.5 g L^−1^ of syringaldehyde (Figure 4). A total of 60 strains (35 lignin-, 13 4-HB-, and 12 syringaldehyde-tolerant strains) from the inhibitor plates were randomly isolated and screened for growth on defined glucose-containing media supplemented with 2.0 g L^−1^ lignin, 0.5 g L^−1^ 4-HB, and 0.5 g L^−1^ syringaldehyde, respectively, in flask cultivations. The spontaneous mutants selected that exhibited robust growth on lignin, 4-HB, and syringaldehyde were termed strains MITXM-61^L33^, MITXM-61^H6^, and MITXM-61^S1^, respectively. We examined the growth kinetics of MITXM-61^L33^, MITXM-61^H6^, and MITXM-61^S1^ on media containing lignin (0 to 4.0 g L^−1^), 4-HB (0 to 1.0 g L^−1^), and syringaldehyde (0 to 0.75 g L^−1^). When 2.0 g L^−1^ lignin, 0.5 g L^−1^ 4-HB, and 0.5 g L^−1^ syringaldehyde were added in the medium, the cell growth of the parental strain MITXM-61 after 2 days of cultivation was almost completely inhibited (Figure 1A,E,F). By contrast, lignin-tolerant strain MITXM-61^L33^, 4-HB-tolerant strain MITXM-61^H6^, and syringaldehyde-tolerant strain MITXM-61^S1^ were able to grow on media containing up to 3.0 g L^−1^ lignin, 1.0 g L^−1^ 4-HB, and 0.5 g L^−1^ syringaldehyde, respectively, after 2 days of cultivation (Figure 5A,B,C).

### Adaptive evolution of lignin/4-HB/syringaldehyde-tolerant *R. opacus* strains

**Figure 6:**
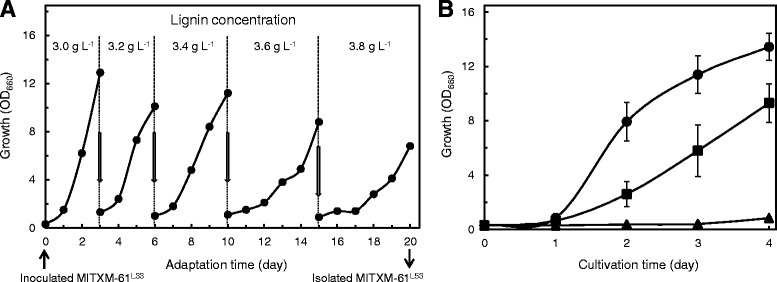
Construction of lignin-tolerant *R. opacus* strains. **(A)** Adaptive evolution of strain MITXM-61^L33^ for improved lignin tolerance. The strain was grown in a defined medium containing 16 g L^−1^ glucose supplemented with different concentrations of lignin (3.0 ~ 3.8 g L^−1^) in a flask. Five milliliters of the culture were sequentially transferred into a flask containing 50 ml of the fresh medium after 3, 6, 10, and 15 days of cultivation. **(B)** Growth of evolved *R. opacus* strains in the presence of lignin. Strains MITXM-61^L53^ (black circle), MITXM-61^L33^ (black square), and MITXM-61 (black triangle) were grown in a defined medium containing 16 g L^−1^ glucose supplemented with 3.5 g L^−1^ lignin. Values and error bars represent the mean and the standard deviation of triplicate experiments.

Strain MITXM-61 was able to adapt to lignin, 4-HB, and syringaldehyde by incubating on plates in the presence of sublethal concentrations of each compounds as described above (Figures 4 and 5). We developed *R. opacus* strains with tolerances to lignin, 4-HB, and syringaldehyde through a directed evolution strategy. Lignin-tolerant strain MITXM-61^L33^ was subjected to an adaptive evolution procedure for further improvement of its lignin tolerance. Cells of MITXM-61^L33^, at an initial optical density (OD)_660_ of 0.3, were added to a defined glucose-containing medium with 3.0 g L^−1^ lignin, and the culture was allowed to grow until early stationary phase in a flask. A 10% (v v^−1^) inoculum from a previous culture was sequentially transferred to the fresh medium containing increased concentrations of lignin at 3.2, 3.4, 3.6, and 3.8 g L^−1^ after 3, 6, 10, and 15 days of cultivation. The growth performance in the evolutionary progress is presented in Figure 6A. After the adaptive evolution at 3.8 g L^−1^ lignin, the culture was streaked on defined medium for isolated colonies. One of the colonies was chosen and termed MITXM-61^L53^, following comparison of 25 colonies for their growth in the defined lignin-containing medium. Figure 6B shows the growth curves of two lignin-tolerant strains MITXM-61^L53^ and MITXM-61^L33^, and the parental strain MITXM-61 in defined liquid medium containing 3.5 g L^−1^ lignin. No growth of strain MITXM-61 was observed over 4 days of culture. While the two tolerant strains were able to grow in the presence of the lignin concentration tested, the evolved strain MITXM-61^L53^ exhibited overall improved growth and a higher final cell density as compared with strain MITXM-61^L33^.

We next generated *R. opacus* strains that are double-tolerant to lignin and 4-HB. The competent cells of the lignin-tolerant strain MITXM-61^L53^ were treated by electroporation, and plated on a defined glucose-containing agar medium with 0.75 g L^−1^ 4-HB. Ten colonies from the plate were isolated after 14 days of cultivation, and cultivated in a defined glucose-containing medium with 0.75 g L^−1^ 4-HB. Strain MITXM-61^HL6^ exhibited the most robust cell growth in the flask culture, reaching an OD_660_ of approximately 10 after 3 days of cultivation. To further improve the 4-HB tolerance in strain MITXM-61^HL6^, a 10% (v v^−1^) inoculum from the previous batch-culture was sequentially transferred to the medium with increasing 4-HB concentrations at 0.8, 0.85, 0.9, and 0.95 g L^−1^. This transfer procedure was repeated for four iterations. The cell growth in the adaptation is shown in Additional file 1: Figure S1A. The cells after 20 days of cultivation were plated on defined medium for isolated colonies. The growth of 20 isolates was examined in defined 4-HB-containing liquid medium, and designated MITXM-61^HL27^ was selected as a strain exhibiting the highest tolerance to 4-HB. Strain MITXM-61^HL27^ showed the enhanced cell growth on the defined glucose-containing medium with 0.75 g L^−1^ 4-HB relative to strain MITXM-61^HL6^ (Additional file 1: Figure S1B).

We then constructed strains that are triple-tolerant to lignin, 4-HB, and syringaldehyde. The pulsed cells of lignin- and 4-HB-tolerant strain MITXM-61^HL27^ were plated on medium containing 1.5 g L^−1^ syringaldehyde. Twenty colonies were isolated, and one of the fastest growing isolates, MITXM-61^SHL18^, was selected. To additively enhance syringaldehyde-tolerance in MITXM-61^SHL18^, sequential transfers of the cells in batch flask cultures with defined medium containing increasing concentrations of syringaldehyde at 0.5, 0.55, 0.6, 0.65, and 0.7 g L^−1^ were carried out. This transfer procedure was repeated for four iterations over a duration of 20 days. The growth performance during the progress of the evolution is presented in Additional file 1: Figure S2A. Sixteen colonies from the culture of the fifth generation were randomly isolated, and designated MITXM-61^SHL33^ was chosen after comparing the isolates for their growth in the defined syringaldehyde-containing medium. The evolved strain MITXM-61^SHL33^ on the defined glucose-containing medium with 0.6 g L^−1^ syringaldehyde exhibited improved cell growth with a shorter lag phase as compared to the derivative strain MITXM-61^SHL18^ (Additional file 1: Figure S2B).

### Growth behavior of the evolved lignin/4-HB/syringaldehyde-tolerant strain

To validate whether the evolved lignin/4-HB/syringaldehyde-tolerant strain MITXM-61^SHL33^ had a growth advantage over strain MITXM-61 in the presence of lignocellulose-derived inhibitors, we compared the growth profiles of both strains in refined sugar-defined media containing the inhibitors and genuine lignocellulosic hydrolysates. IC_50_s, which indicate the concentrations of lignin, furfural, HMF, vanillin, 4-HB, syringaldehyde, levulinic acid, formic acid, and acetic acid in defined glucose-containing liquid media that cause 50% growth inhibition, were determined for strain MITXM-61^SHL33^. As shown in Table 1, the IC_50_ values of MITXM-61^SHL33^ and MITXM-61 for six of the inhibitors tested, except for lignin, 4-HB, and syringaldehyde, were almost identical to one another. The IC_50_s of lignin, 4-HB, and syringaldehyde in strain MITXM-61^SHL33^ were 3.5 (±0.26) g L^−1^, 0.73 (±0.03) g L^−1^, and 0.57 (±0.01) g L^−1^, respectively, and were 3-fold, 2.5-fold, and 2-fold higher, respectively, than those of strain MITXM-61, suggesting that the tolerance to lignin, 4-HB, and syringaldehyde of strain MITXM-61^SHL33^ was improved as compared to that of the parental strain MITXM-61.

**Figure 7:**
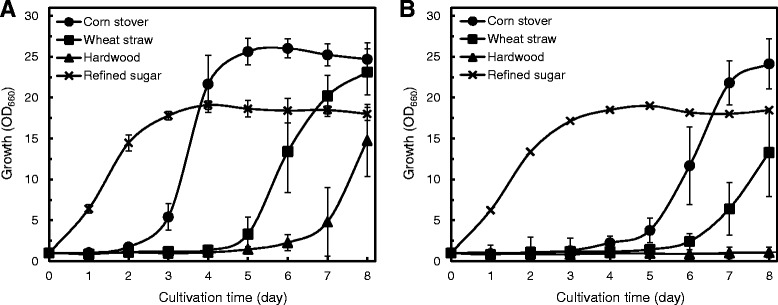
Growth behavior of the evolved lignin/4-HB/syringaldehyde-tolerant *R. opacus* strain on lignocellulosic hydrolysates. The evolved strain MITXM-61^SHL33^ **(A)** and the parental strain MITXM-61 **(B)** were grown in corn stover, wheat straw, and hardwood hydrolysates contained 50.0 g L^−1^ total sugars supplemented with 1 g L^−1^ (NH_4_)_2_SO_4_ (adjusted to pH 7.0 with 5 M NaOH), and a modified defined medium containing mixed refined sugars comprised of 31.2 g L^−1^ glucose, 17.0 g L^−1^ xylose, and 1.8 g L^−1^ arabinose supplemented with 1 g L^−1^ (NH_4_)_2_SO_4_. Values and error bars represent the mean and the standard deviation of triplicate experiments.

We next explored the impact of cell growth of strain MITXM-61^SHL33^ on real lignocellulosic hydrolysates composed of 50 g L^−1^ total sugars from corn stover, wheat straw, and hardwood contained growth inhibitors (Additional file 1: Figure S3). The growth performance of strains MITXM-61^SHL33^ and MITXM-61 grown in the refined sugar-defined medium in the absence of any inhibitors, as a control, reached the stationary phase at the maximum OD_660_ of 17 to 18 within 3 days post-inoculation with no significant difference in the growth rate. In contrast, cultivating in the hydrolysates, the cell growth of strain MITXM-61^SHL33^ started after 3 days on corn stover, 5 days on wheat straw, and 7 days on hardwood (Figure 7A), while strain MITXM-61 showed considerably longer lag phases and started growing after 5 days on corn stover and 7 days on wheat straw, and was not able to grow on hardwood during 8 days (Figure 7B). Strain MITXM-61^SHL33^ exhibited a significantly reduced lag phase and allowed growth in the hydrolysates, media that suppressed the growth of strain MITXM-61. Furthermore, both strains were grown in refined sugar-defined media containing 50 g L^−1^ total sugars supplemented with or without nine representative inhibitors at their concentrations found in the hydrolysates. The lag phase of strain MITXM-61 on the defined medium in the presence of the nine inhibitors was 3 days longer on the corn stover-based cocktail and 5 to 6 days longer on wheat straw and hardwood-based cocktails than in the absence of inhibitors (Additional file 1: Figure S4B). Meanwhile, the growth profile of strain MITXM-61^SHL33^ on the defined medium in the presence of the nine inhibitors was almost the same as corn stover-based cocktail and a 1 to 2 day longer lag phase on wheat straw and hardwood-based cocktails compared to the medium in the absence of inhibitors (Additional file 1: Figure S4A). These findings support that the evolved strain MITXM-61^SHL33^ gained enhanced lignin/4-HB/syringaldehyde tolerances and exhibited an advantage in fermentations on actual lignocellulosic hydrolysates, compared to the parental strain MITXM-61.

## Discussion

A recently engineered, TAG-producing strain of *R. opacus* MITXM-61 exhibited a xylose-fermenting feature with the capability of utilizing mixed sugars of glucose and xylose at high concentrations in genuine lignocellulosic hydrolysate and producing a large amount of intracellular TAGs as a precursor for lipid-based biofuels [56]. However, MITXM-61 was shown to be sensitive to certain compounds present in these hydrolysates that interfere with the growth and metabolic activity of microbial cells, resulting in low yield and productivity in fermentation processes using this strain. A more in-depth understanding of the toxic effects of lignocellulose-derived materials is critical to improving sugar utilization capabilities and product yields in the process. In the present study, it was demonstrated that common lignocellulose-derived materials undoubtedly inhibited the cell growth and TAG production of *R. opacus* MITXM-61, and tolerance of the strain to certain inhibitors could be improved *via* adaptive evolution.

In general, lignocellulose-derived inhibitors can be categorized into phenols, furans, and organic acids [20-22]. We compared the effects of the nine common inhibitors, lignin, vanillin, 4-HB, syringaldehyde, furfural, HMF, levulinic acid, formic acid, and acetic acid, on the growth and TAG production of strain MITXM-61. As expected, all materials tested inhibited the cell growth of *R. opacus*, but there were significant differences in the inhibition of growth rates and final cell densities in the presence of each individual material (Figure 1 and Table 1). The phenols and furans showed potent inhibitory effects at a concentration of 1 g L^−1^, whereas organic acids had scant impacts at a concentration of up to 2 g L^−1^. The lipid content of the cells grown in the presence of <IC_50_ of each individual material was nearly identical to that in the absence of any inhibitors, suggesting that lipid biosynthesis pathways in the strain remained relatively intact (Figure 2). According to our findings, the inhibitory materials apparently suppress cell growth more aggressively than lipid biosynthesis in MITXM-61. Among the organic acids tested, interestingly, levulinic acid and acetic acid stimulated the cell growth and lipid biosynthesis of MITXM-61 at concentrations similar to the IC_50_. Furthermore, the combination of the common lignocellulose-derived inhibitors caused greater growth suppression and synergistic inhibitory effects on cell growth of MITXM-61 than the sum of all inhibitions caused by the individual compounds (Figure 3). The inhibition profiles and physiological responses of MITXM-61 to those inhibitors were in reasonable agreement with the findings for other oleaginous microorganisms [30,32,34,58,60,61]. It was notable that levulinic acid could affect the lipid biosynthesis pathway of *R. opacus* and change the fatty acids composition of the TAG molecule (Tables 2 and 3). In most cases, lignocellulose-derived organic acids have no significant influence on the fatty acid component of microbial TAG molecules [32,34]. Interestingly, the addition of levulinic acid, at its IC_50_, into the medium altered the principal fatty acid component of *Rhodococcus* TAGs: from even-numbered carbon chain (C16 and C18 series) fatty acids synthesized in the absence of levulinic acid to odd-numbered carbon chain (C15 and C17 series) fatty acids.

We also attempted to construct *R. opacus* strains with improved tolerance to lignocellulose-derived inhibitors. It has been shown that adaptive evolution of yeast cells to lignocellulose-derived inhibitors is an effective approach for improving the tolerance and fermentation performance [51,62]. In the current study, this strategy proved to be valuable for improvement of *R. opacus* tolerance to the inhibitors even when the genetic constitution is unidentified. We devised an evolutionary adaptation approach using short-term serial transfers of the cell cultures in inhibitor-containing liquid media following spontaneous adaptation of *R. opacus* cells on defined agar media with single inhibitors. The resulting strain, MITXM-61^SHL33^, exhibited multiple enhanced resistances to materials such as lignin, 4-HB, and syringaldehyde (Table 1). It also showed significant improved fermentation performance in the defined medium with the addition of those inhibitors, and all three lignocellulosic hydrolysates, as compared to that of strain MITXM-61 (Additional file 1: Figure S4, Figure 7). However, the evolved MITXM-61^SHL33^ strain grown in lignocellulosic hydrolysates, especially in hardwood hydrolysate, clearly exhibited a longer lag phase as compared to growth in defined media containing equivalent amounts of the nine inhibitors found in the lignocellulosic hydrolysate (Figure 7A, Additional file 1: Figure S4A). This indicates that different materials released during treatment, other than the nine common compounds focused on in this study, may result in an inhibition of the fermentation performance of *R. opacus*.

High-cell-density cultivation is necessary to maximize volumetric productivity and minimize the cost of microbial fermentations [63,64]. The feedstocks used should consist of highly concentrated sugars, but with increasing the sugar content in lignocellulosic hydrolysates, there will ultimately be increased amounts of inhibitors. The content and composition of inhibitors in the hydrolysates are widely varied among conditions used during pretreatment, due to combined factors that include the raw materials (type, age, and harvest time of lignocellulose) and the operational conditions (reaction temperature, pH, time, and catalyst concentrations) employed for hydrolysis [13,23,24,47]. It is also well known that the fermentative performances in hydrolysates and related inhibitors depend on the microorganism used [25,26,65]. Indeed, corn stover, wheat straw, and hardwood hydrolysate used in this study did not show any inhibitory effects on the growth and ethanol production of *S. cerevisiae* (data not shown). *R. opacus* MITXM-61 appears to exhibit lower tolerance toward pretreatment-generated inhibitors as compared to yeasts such as *Saccharomyces*, *Rhodosporidium*, *Pichia*, and *Candida* [21,32,66] and bacteria such as *Corynebacterium*, *Escherichia*, and *Zymomonas* [27-29]. Very little is known about the inhibitory effect on *R. opacus* of specific lignocellulose-derived materials. Further investigation is needed to determine whether some of the other materials have synergistic effects on cell growth inhibition. A combination of the inhibitor-tolerant *R. opacus* strain with desired properties for detoxification of lignocellulosic hydrolysates will likely improve the robustness of lignocellulose-to-TAG processes. Additionally, Kosa *et al*. have recently reported that *R. opacus* strains can convert lignin and its related compounds to TAGs in extremely low concentrations with no growth inhibition [67,68]. The development of MITXM-61^SHL33^ provides a critical step toward generating a cost-effective bioprocess for advanced lignocellulosic biofuels.

## Conclusions

This is the first comprehensive study that investigated the effects of inhibitors commonly generated during the pretreatment of lignocellulosic biomass on the growth of TAG-producing *R. opacus* strains and demonstrated the potential for improving tolerance to lignocellulose-derived inhibitors. Fermentation in the presence of lignocellulose-derived inhibitors resulted in poor cell growth and increased times to reach the maximum TAG production levels. Out of the nine common lignocellulose-derived inhibitors studied, 4-HB, syringaldehyde, vanillin, furfural, HMF, and lignin had potent inhibitory effects at a concentration of 1 g L^−1^.

An evolutionary adaptation was developed to alleviate the effect of lignocellulose-derived inhibitors in *R. opacus*. We successfully constructed a strain, MITXM-61^SHL33^, with improved tolerance to lignin, 4-HB, and syringaldehyde. The evolved strain had a growth advantage over the parental strain in inhibitor-containing synthetic media and also in genuine lignocellulosic hydrolysates. Such knowledge will be useful for the development and optimization of the lignocellulosic biomass pretreatment method and for generation of *R. opacus* strains with increased inhibitor tolerance in the industrial TAG production.

## Methods

### Materials

Hydrolysates from corn stover, wheat straw, and hardwood using dilute-acid pretreatment were kindly provided by Sweetwater Energy Inc. (Rochester, NY, USA). The corn stover contained 34.4 g L^−1^ glucose, 13.9 g L^−1^ xylose, and 1.7 g L^−1^ arabinose with 2.9 g L^−1^ lignin, 0.002 g L^−1^ furfural, 0.016 g L^−1^ HMF, 0.007 g L^−1^ vanillin, 0.002 g L^−1^ 4-HB, 0.070 g L^−1^ syringaldehyde, 0.009 g L^−1^ levulinic acid, 0.023 g L^−1^ formic acid, and 0.329 g L^−1^ acetic acid (Additional file 1: Figure S3C, D). The wheat straw was composed of 30.4 g L^−1^ glucose, 18.1 g L^−1^ xylose, and 1.5 g L^−1^ arabinose with 5.1 g L^−1^ lignin, 0.001 g L^−1^ furfural, 0.026 g L^−1^ HMF, 0.009 g L^−1^ vanillin, 0.002 g L^−1^ 4-HB, 0.066 g L^−1^ syringaldehyde, 0.018 g L^−1^ levulinic acid, 0.090 g L^−1^ formic acid, and 1.240 g L^−1^ acetic acid (Additional file 1: Figure S3E, F). The hardwood was comprised of 29.0 g L^−1^ glucose, 18.9 g L^−1^ xylose, and 2.1 g L^−1^ arabinose with 4.2 g L^−1^ lignin, 0.001 g L^−1^ furfural, 0.026 g L^−1^ HMF, 0.022 g L^−1^ vanillin, 0.004 g L^−1^ 4-HB, 0.057 g L^−1^ syringaldehyde, 0.029 g L^−1^ levulinic acid, 0.097 g L^−1^ formic acid, and 1.309 g L^−1^ acetic acid (Additional file 1: Figure S3G, H). Acetic acid was purchased from EMD chemicals (Gibbstown, NJ, USA). Other chemicals were purchased from Sigma-Aldrich (St. Louis, MO, USA) unless otherwise noted. Lignin was obtained from Sigma-Aldrich #471003 (kraft pine lignin, alkali low sulfonate content, water-soluble, average Mw = 10,000) [69].

### Bacterial strains, media, and cultivation

A list of strains used in this study is given in Additional file 2: Table S1. *R. opacus* MITXM-61 was constructed in a previous study [56]. MITXM-61 derivatives with modified tolerance to lignocellulose-derived inhibitors were constructed in this study.

The culture media used were LB broth (BD Diagnostic Systems, Sparks, MD, USA) and a defined medium containing the following composition per liter: 16 g glucose, 1.0 g (NH_4_)_2_SO_4_ and mineral components consisted of 1.0 g MgSO_4_**·**7H_2_O, 0.015 g CaCl_2_**·**2H_2_O, 1.0 ml of a trace element solution, 1.0 ml stock A solution, and 35.2 ml 1.0 M phosphate buffer as described [54]. Modifications of the defined medium are stated in tables and figure legends. In the case of inhibitor-containing cultures, the compounds were added to the cooled medium as filter (0.2 μm pore size) sterilized stock solutions, which were adjusted to pH 7.0 with 5 M NaOH. Solid media were supplemented with 2% (*w*/*v*) agar. The strains were routinely maintained on LB agar medium and preserved in 20% (*v*/*v*) glycerol at −80°C.

All cultures were grown at 30°C. Submerged cultivations were carried out using 250-ml baffled flasks with a working volume of 50 ml, and incubated on a rotary shaker at 200 rpm (Multitron, Infors, Bottmingen, Switzerland). *R. opacus* seed cultures were prepared in the defined medium. Cells from colonies grown on LB agar medium for 3 days were inoculated into the medium in a flask. The culture was incubated for 2 days until the late exponential growth phase. Unless otherwise stated, cultures for flask experiments were inoculated with the seed culture to an initial OD of 0.3 (7.5 × 10^7^ cfu ml^−1^). The half maximal inhibitory concentrations of the lignocellulose-derived compounds (IC_50_) that cause 50% inhibition of the growth of *R. opacus* were estimated from the growth profiles through a series of experiments as described [29].

### Strain construction

The generation of lignocellulose-derived inhibitor-tolerant *R. opacus* strains was accomplished by short-term serial transfers of the cell cultures to inhibitor-containing liquid media following spontaneous adaptation on defined agar media with the inhibitory compounds.

Competent cells of *R. opacus* were prepared, as previously described [55], and treated with electroporation (Bio-Rad gene pulser, Hercules, CA, USA) at 2.5 kV, 25 μF, and 200 Ω in a 2 mm electroporation cuvette (VWR, Radnor, PA, USA). The pulsed cells were diluted with LB broth, regenerated for 3 h with gentle agitation, plated onto defined agar medium containing the lignocellulose-derived inhibitor, and incubated to select for inhibitor-tolerant strains. After 14 days of cultivation, evolved strains that exhibited robust growth on the inhibitor-containing medium were isolated. The isolates were used for serial transfers of cells using repetitive cultures in flasks. The sequential cultivation was performed in defined inhibitor-containing liquid media with a stepwise increase in concentration of inhibitor compound. Cells grown on LB agar medium for 3 days were inoculated into a 250-ml baffled flask with 50 ml of the defined medium containing the inhibitor at appropriate concentrations. Allowing the growth to reach to the early stationary phase, 5 ml of culture broth from the preceding flask was transferred to a new flask culture with the appropriate medium composition. This procedure was repeated for four iterations. After a total of four generations (20 days), at least ten colonies were randomly isolated by plating for single clones on the defined agar medium and tested again for growth in flasks with the defined medium supplemented with the inhibitor at appropriate concentrations. One of the fastest growing evolved strains was selected and used for further experiments.

### Analytical methods

Cell growth was estimated by determining the optical density (OD) at 660 nm (Thermo Scientific GENESYS 20, Waltham, MA) or the cell dry weight (CDW). The CDW was determined by lyophilizing cell pellet after centrifuging 10 ml of culture broth at 8,000 *g* for 15 min and washing the cell pellet twice in deionized water. The lyophilized cell pellet was used to analyze the fatty acids of TAGs. To determine the fatty acid content of the cells and the composition of lipids, the whole cells were subjected to methanolysis and the resulting fatty acid methyl esters (FAMEs) were analyzed by gas chromatography (GC) as described in detail [54]. The fatty acids were identified and quantified by comparison to standard FAMEs: methyl myristate (C14:0), methyl pentadecanoate (C15:0), methyl palmitate (C16:0), methyl palmitoleate (C16:1), methyl heptadecanoate (C17:0), methyl *cis*-10-heptadecenoate (C17:1), methyl stearate (C18:0), methyl oleate (C18:1), and methy linoleate (C18:2). Fatty acid content was defined as the percentage of the ratio of fatty acids to cell dry weight (% CDW). The supernatants of the culture broth were used for analyses of residual sugars after filtration through syringe filters (0.2 μm pore size). Glucose, xylose, arabinose, formic acid, acetic acid, and levulinic acid concentrations were measured by high-performance liquid chromatography (HPLC, Agilent 1100 system) fitted with an Aminex HPX-87H column (300 × 7.8 mm, BIO-RAD) coupled to a refractive index (RI) detector as previously described [55]. HMF, furfural, 4-HB, vanillin, and syringaldehyde were analyzed using HPLC (Agilent 1260 infinity analytical-scale purification system) fitted with a XTerra MS C18 column (150 × 2.1 mm, Waters). The column was eluted with a gradient of water and acetonitrile, both of which contained 3 mM formic acid, at 25°C and a flow rate of 0.4 ml min^−1^. The gradient design consisted of three steps: (i) 2% (*v*/*v*) acetonitrile was applied for 7 min, (ii) the concentration of acetonitrile was increased linearly to 20% during 28 min, and (iii) 100% acetonitrile was applied for 5 min. Compounds were detected by UV at a wavelength of 284 nm. Samples were filtered through 0.45 μm syringe filters prior to inject into the HPLC system. Soluble lignin content was calculated by using UV absorbance of the filtrate at 205 nm (Agilent 8453 spectrophotometer) [70].

## Additional files

Additional file 1: Figure S1. Construction of 4-HB-tolerant *R. opacus* strains. **(A)** Adaptive evolution of strain MITXM-61^HL6^ for improved 4-HB tolerance. **(B)** Growth of evolved *R. opacus* strains in the presence of 4-HB. **Figure S2.** Construction of syringaldehyde-toletant *R. opacus* strains. **(A)** Adaptive evolution of strain MITXM-61^SHL18^ for improved syringaldehyde tolerance. **(B)** Growth of evolved *R. opacus* strains in the presence of syringaldehyde. **Figure S3.** HPLC-chromatograms of lignocellulose-derived inhibitors and hydrolysates from corn stover, wheat straw and hardwood. **Figure S4.** Growth of the evolved lignin/4-HB/syringaldehyde-tolerant strain on defined media containing inhibitors found in lignocellulosic hydrolysates. The evolved strain MITXM-61^SHL33^ **(A)** and the parental strain MITXM-61 **(B)** were grown in modified defined media containing mixed refined sugars supplemented with or without equivalent amounts of the nine common inhibitors detected in hydrolysates. Additional file 2: Table S1. Bacterial strains used in this study.

## Abbreviations

4-HB: 4-hydroxybenzaldehyde
CDW: cell dry weight
FAME: fatty acid methyl ester
GC: gas chromatography
HMF: 5-(hydroxymethyl)-2-furaldehyde
HPLC: high-performance liquid chromatography
OD: optical density
TAG: triacylglycerol
