# Supplementary material for: Tolerance and adaptive evolution of triacylglycerol-producing Rhodococcus opacus to lignocellulose-derived inhibitors
Source: Biotechnol Biofuels. 2015 May 13;8:76. doi: 10.1186/s13068-015-0258-3 (PMC4456722; doi:10.1186/s13068-015-0258-3)
Supplement: Additional file 1: Figure S1. — Construction of 4-HB-tolerant R. opacus strains. (A) Adaptive evolution of strain MITXM-61HL6 for improved 4-HB tolerance. (B) Growth of evolved R. opacus strains in the presence of 4-HB. Figure S2. Construction of syringaldehyde-toletant R. opacus strains. (A) Adaptive evolution of strain MITXM-61SHL18 for improved syringaldehyde tolerance. (B) Growth of evolved R. opacus strains in the presence of syringaldehyde. Figure S3. HPLC-chromatograms of lignocellulose-derived inhibitors and hydrolysates from corn stover, wheat straw and hardwood. Figure S4. Growth of the evolved lignin/4-HB/syringaldehyde-tolerant strain on defined media containing inhibitors found in lignocellulosic hydrolysates. The evolved strain MITXM-61SHL33 (A) and the parental strain MITXM-61 (B) were grown in modified defined media containing mixed refined sugars supplemented with or without equivalent amounts of the nine common inhibitors detected in hydrolysates. [file 13068_2015_258_MOESM1_ESM.pdf]

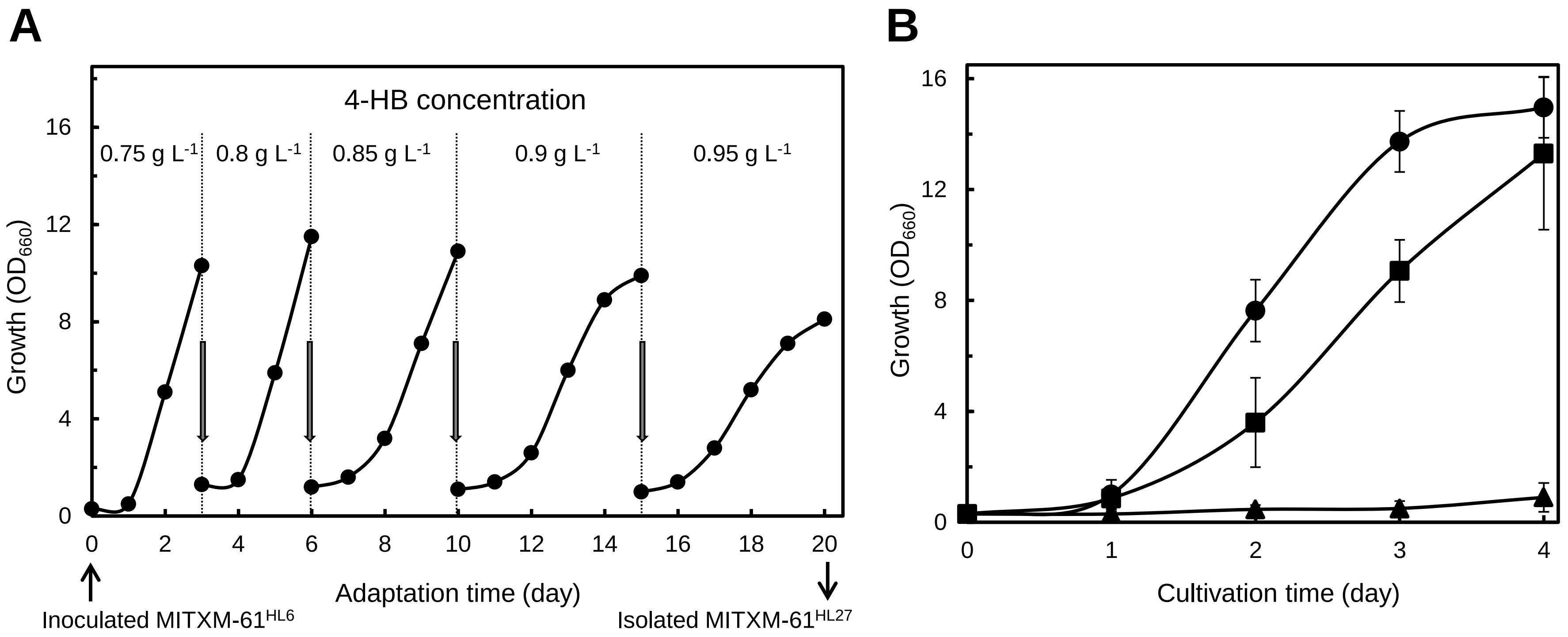

Figure S1 Construction of 4-HB-tolerant *R. opacus* strains. (A) Adaptive evolution of strain MITXM-61^{HL6} for improved 4-HB tolerance. The strain was grown in a defined medium containing 16 g L^{-1} glucose supplemented with different concentrations of 4-HB (0.75~0.95 g L^{-1}) in a flask. Five milliliters of the culture were sequentially transferred into a flask containing 50 ml of the fresh medium after 3, 6, 10 and 15 days of cultivation. **(B)** Growth of evolved *R. opacus* strains in the presence of 4-HB. Strains MITXM-61^{HL27} (●), MITXM-61^{HL6} (■) and MITXM-61^{L53} (▲) were grown in a defined medium containing 16 g L^{-1} glucose supplemented with 0.75 g L^{-1} 4-HB. Values and error bars represent the mean and the standard deviation of triplicate experiments.

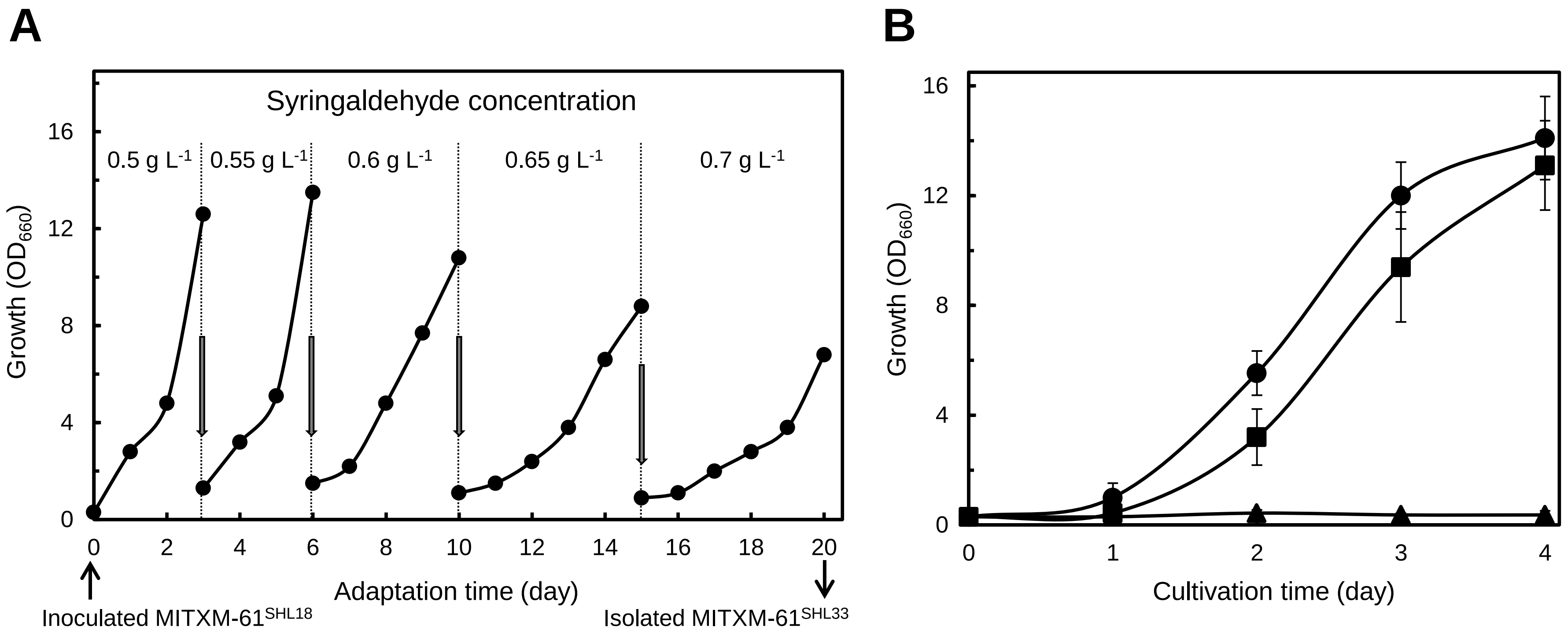

Figure S2 Construction of syringaldehyde-toletant *R. opacus* strains. (A) Adaptive evolution of strain MITXM-61^{SHL18} for improved syringaldehyde tolerance. The strain was grown in a defined medium containing 16 g L^{-1} glucose supplemented with different concentrations of syringaldehyde (0.6~0.8 g L^{-1}) in a flask. Five milliliters of the culture were sequentially transferred into a flask containing 50 ml of the fresh medium after 3, 6, 10 and 15 days of cultivation. **(B)** Growth of evolved *R. opacus* strains in the presence of syringaldehyde. Strains MITXM-61^{SHL33} (●), MITXM-61^{SHL18} (■) and MITXM-61^{HL27} (▲) were grown in a defined medium containing 16 g L^{-1} glucose supplemented with 0.6 g L^{-1} syringaldehyde. Values and error bars represent the mean and the standard deviation of triplicate experiments.

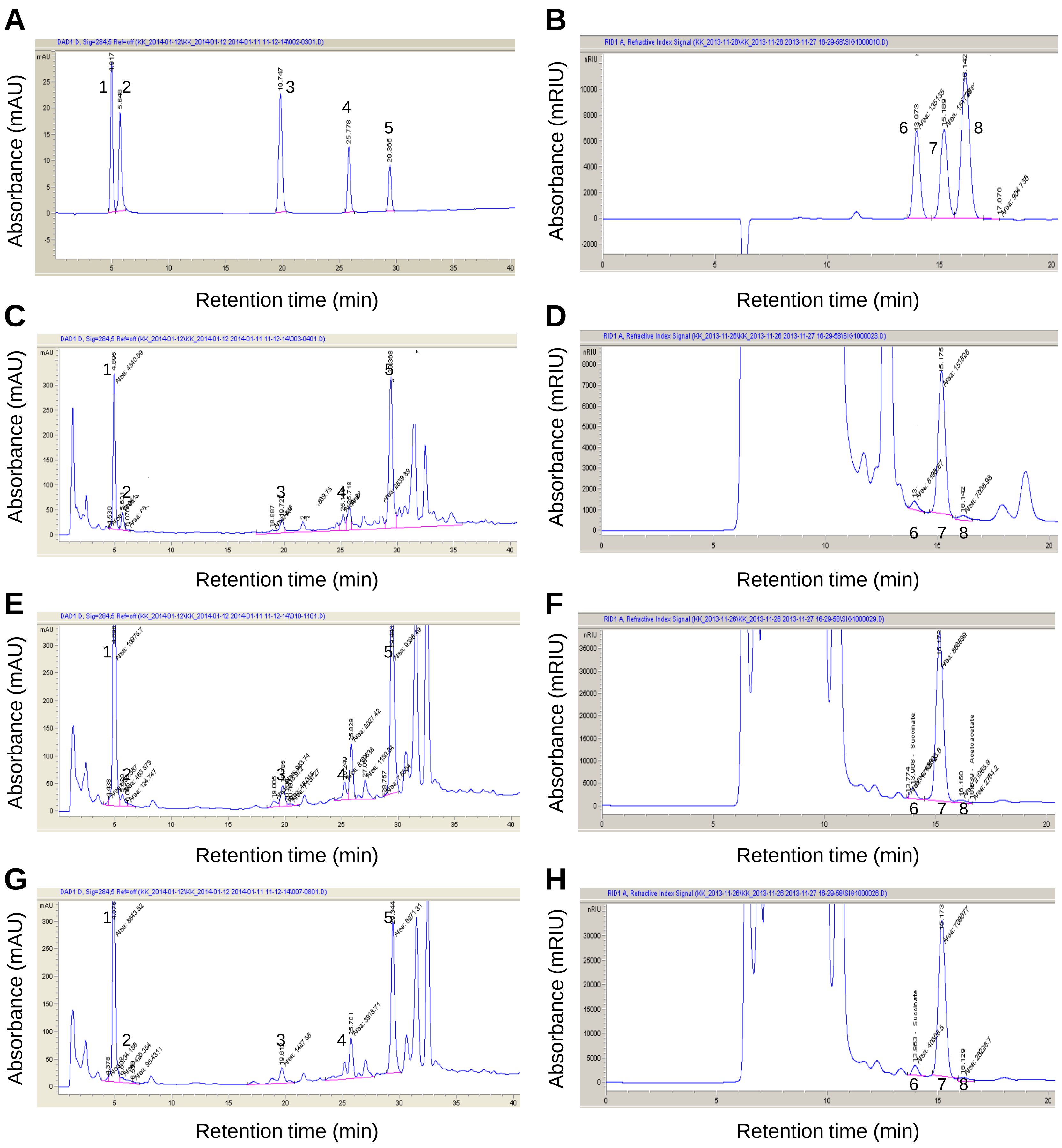

Figure S3 HPLC-chromatograms of lignocellulose-derived inhibitors (A, B) and hydrolysates from corn stover (C, D), wheat straw (E, F) and hardwood (G, H). See the text for details. Peak identifications are as follows: 1, HMF; 2, furfural; 3, 4-HB; 4, vanillin; 5, syringaldehyde; 6, formic acid; 7, acetic acid; 8, levulinic acid.

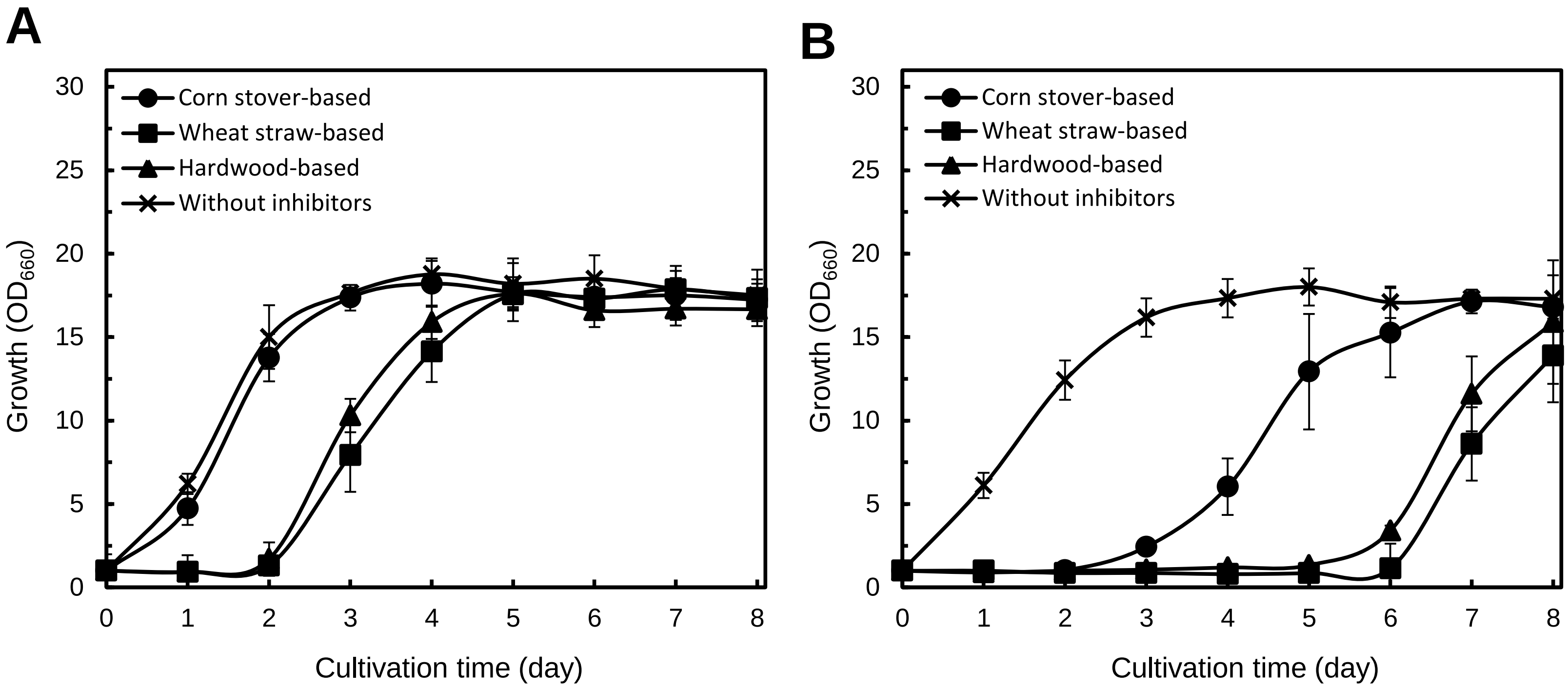

Figure S4 Growth of the evolved lignin/4-HB/syringaldehyde-tolerant strain on defined media containing inhibitors found in lignocellulosic hydrolysates. The evolved strain MITXM-61^{SHL33} **(A)** and the parental strain MITXM-61 **(B)** were grown in modified defined media containing 1 g L^{-1} $(NH_4)_2SO_4$ and mixed refined sugars comprised of 31.2 g L^{-1} glucose, 17.0 g L^{-1} xylose and 1.8 g L^{-1} arabinose supplemented with or without equivalent amounts of the nine common inhibitors detected in corn stover, wheat straw and hardwood hydrolysates. Values and error bars represent the mean and the standard deviation of triplicate experiments.
