# Supplementary material for: Tolerance and adaptive evolution of triacylglycerol-producing Rhodococcus opacus to lignocellulose-derived inhibitors
Source: Biotechnol Biofuels. 2015 May 13;8:76. doi: 10.1186/s13068-015-0258-3 (PMC4456722; doi:10.1186/s13068-015-0258-3)
Supplement: Additional file 2: Table S1. — Bacterial strains used in this study. [file 13068_2015_258_MOESM2_ESM.pdf]

Table S1 Bacterial strains used in this study

Strain	Description	Reference
Rhodococcus opacus		
MITXM-61	Single-colony isolate of strain Xsp8C, evolved after plate cultivation on xylose	[56]
MITXM-61^{L33}	Single-colony isolate of MITXM-61, evolved after plate cultivation on lignin	This study
MITXM-61^{H6}	Single-colony isolate of MITXM-61, evolved after plate cultivation on 4-HB	This study
MITXM-61^{S1}	Single-colony isolate of MITXM-61, evolved after plate cultivation on syringaldehyde	This study
MITXM-61^{L53}	Single-colony isolate of MITXM-61^{L33}, evolved after sequential batch cultivation on lignin	This study
MITXM-61^{HL6}	Single-colony isolate of MITXM-61^{L53}, evolved after plate cultivation on 4-HB	This study
MITXM-61^{HL27}	Single-colony isolate of MITXM-61^{HL6}, evolved after sequential batch cultivation on 4-HB	This study
MITXM-61^{SHL18}	Single-colony isolate of MITXM-61^{HL27}, evolved after plate cultivation on syringaldehyde	This study
MITXM-61^{SHL33}	Single-colony isolate of MITXM-61^{SHL18}, evolved after sequential batch cultivation on syringaldehyde	This study
